# The prostate cancer risk variant rs55958994 regulates multiple gene expression through extreme long-range chromatin interaction to control tumor progression

**DOI:** 10.1126/sciadv.aaw6710

**Published:** 2019-07-17

**Authors:** Yuyang Qian, Lei Zhang, Mingyang Cai, Hongxia Li, Heming Xu, Hongzhen Yang, Zhongfang Zhao, Suhn Kyong Rhie, Peggy J. Farnham, Jiandang Shi, Wange Lu

**Affiliations:** 1 State Key Laboratory of Medicinal Chemical Biology and College of Life Sciences, Nankai University, 94 Weijin Road, 300071 Tianjin, China.; 2 Department of Stem Cell Biology and Regenerative Medicine, Broad Center for Regenerative Medicine and Stem Cell Research, Keck School of Medicine, University of Southern California, Los Angeles, CA 90033, USA.; 3 Department of Biochemistry and Molecular Medicine, Norris Comprehensive Cancer Center, Keck School of Medicine, University of Southern California, Los Angeles, CA 90033, USA.

## Abstract

Genome-wide association studies identified single-nucleotide polymorphism (SNP) rs55958994 as a significant variant associated with increased susceptibility to prostate cancer. However, the mechanisms by which this SNP mediates increased risk to cancer are still unknown. In this study, we show that this variant is located in an enhancer active in prostate cancer cells. Deletion of this enhancer from prostate tumor cells resulted in decreased tumor initiation, tumor growth, and invasive migration, as well as a loss of stem-like cells. Using a combination of capture chromosome conformation capture (Capture-C) and RNA sequencing, we identified genes on the same and different chromosomes as targets regulated by the enhancer. Furthermore, we show that expression of individual candidate target genes in an enhancer-deleted cell line rescued different aspects of tumorigenesis. Our data suggest that the rs55958994-associated enhancer affects prostate cancer progression by influencing expression of multiple genes via long-range chromatin interactions.

## Teaser

The rs55958994-associated enhancer regulates prostate cancer progression by long-range regulation of multiple target genes.

## INTRODUCTION

Prostate cancer (PCa) is the most commonly diagnosed cancer in aging men, particularly in developed countries (*1*). Despite advances in treatment, PCa is still one of the leading causes of cancer death worldwide. Conventional PCa therapies include surgery, radiation, hormonal ablation, and chemotherapy (*2*). These therapies fail likely because of the existence of cancer stem cells (CSCs), which leads to relapse, metastasis, and resistance to conventional therapies (*3*, *4*).

Genome-wide association studies coupled with large-scale collaborative replication efforts have led to the identification of more than 100 PCa risk variants (*5*–*13*). In a fine-mapping study, the single-nucleotide polymorphism (SNP) rs55958994 was identified as one of the most significant variants at the chromosome 12q13 (*14*, *15*) risk locus that is associated with increased susceptibility to PCa. This risk SNP is located in the first intron of the *KRT8* gene. The mechanism as to how rs55958994 confers PCa risk and progression is still unknown.

Chromatin three-dimensional architecture studies show that regulatory elements can activate or repress gene expression through long-range chromatin interactions (*16*–*18*). In addition, risk SNPs located in noncoding genomic regions have been shown to function by regulating expression of long-range genes (*16*, *19*–*21*). For example, the risk SNPs rs1447295, rs16901979, and rs6983267 in enhancers located in the well-documented PCa risk region at 8q24 can regulate *MYC* expression (*22*, *23*). The deletion of H3K27ac peak region (chr8: 128412627 to 128415224) that harbors colon cancer risk SNPs induced expression alterations of several genes, including the *MYC* oncogene (*19*, *24*).

Although the connection between risk SNPs, MYC, and cancer is well studied, in many cases, the genes suggested to be regulated by risk SNPs have not been demonstrated to be involved in tumorigenesis. Here, we demonstrate that the risk SNP rs55958994 is located in a putative active enhancer. To uncover the molecular mechanisms by which this risk-associated enhancer contributes to PCa progression, we performed capture chromosome conformation capture (Capture-C) to reveal the interactome of the risk enhancer and conducted RNA sequencing (RNA-seq) to measure gene expression changes after the deletion of the enhancer region using the CRISPR-Cas9 system. Risk enhancer–deleted cells exhibited reduced tumor initiation, tumor growth, invasive migration, and a number of CSCs. Multiple genes were identified as target genes of the risk enhancer. We showed that the introduction of these candidate genes individually into the enhancer-deleted cells can rescue one or multiple aspects of tumorigenesis. We suggest that the enhancer-containing PCa risk SNP rs55958994 contributes to PCa tumorigenesis, metastasis, and CSC population by regulating multiple genes through long-range chromatin interactions.

## RESULTS

### The risk SNP rs55958994 resides within a functional enhancer element in PCa cells

**Fig. 1:**
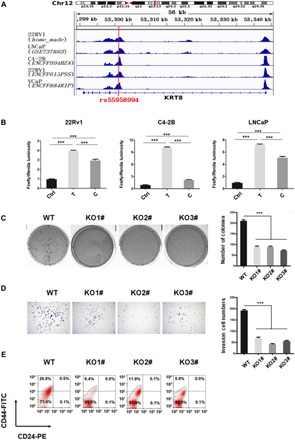
The chromatin locus containing rs55958994 is a functional enhancer in PCa cells. (**A**) H3K27ac ChIP-seq data from PCa cell lines are shown. (**B**) Luciferase reporter activity in 22Rv1, C4-2B, and LNCaP cells transfected with luciferase reporters. Ctrl, luciferase reporter without the inserted enhancer; C, luciferase reporter with the rs55958994-associated enhancer region with the nonrisk allele “(C)”; T, luciferase reporter with the rs55958994-associated enhancer region with the risk allele (T). Data represent means ± SEM of three independent experiments. ***P < 0.001. (**C**) Soft agar colony formation assays comparing wild-type (WT) 22Rv1 cells and three enhancer-deleted cell lines [knockouts (KOs) 1 to 3]. Quantification is at right. Data represent means ± SEM of three independent experiments. ***P < 0.001. (**D**) Transwell assays in WT 22Rv1 cells and three enhancer-deleted cell lines (KOs 1 to 3). Cells that had migrated to lower chambers were stained with 0.1% crystal violet. Quantification is at right. Data represent means ± SEM of three independent experiments. ***P < 0.001. (**E**) Fluorescence-activated cell sorting (FACS) analysis of the cell surface markers CD44 and CD24 in WT 22Rv1 cells and three enhancer-deleted cell lines (KOs 1 to 3). FITC, fluorescein isothiocyanate; PE, phycoerythrin.

SNP rs55958994, located in the first intron of the *KRT8* gene, is one of the most significant PCa risk–associated genetic variants. To understand the function of this noncoding SNP, we used chromatin immunoprecipitation (ChIP) followed by next-generation sequencing (ChIP-seq) for the active histone mark H3K27ac to identify enhancer-like elements in the 22Rv1 PCa cell line. We observed significant enrichment of H3K27ac signals at the risk SNP locus, indicating that the noncoding region containing the risk SNP is a putative active enhancer (Fig. 1A). H3K27ac profiles of other PCa cell lines from the ENCODE (Encyclopedia of DNA Elements) database (*25*), including LNCaP, C4-2B, VCaP, and PC3, also showed enrichment of this active histone mark at the rs55958994-associated region, suggesting that this region could be an active enhancer in multiple PCa cell lines. We note that this H3K27Ac site is the only enhancer region that is marked by any of the fine-mapped PCa risk SNPs at 12q13. This single H3K27Ac site overlaps with two risk SNPs, rs137898974 and rs55958994 (fig. S1). SNP rs137898974 is in the exon region of *KRT8*, while rs55958994 is in the *KRT8* intron region. Because it would be difficult to study the function of the enhancer without destroying the integrity of the *KRT8* coding region, we have focused on rs55958994 in our studies.

To determine whether the risk (T) allele of rs55958994 influences enhancer activity, as compared to the nonrisk (C) allele, we cloned fragments corresponding to either allele into a luciferase promoter reporter and compared their activity in 22Rv1, C4-2B, and LNCaP cells. We found that both alleles enhanced promoter activity, with a stronger effect from the risk (T) allele than from the nonrisk allele (C) (Fig. 1B). This result supports the hypothesis that rs55958994 influences enhancer activity in PCa cells. Furthermore, ChIP-seq data from the ENCODE database (*25*) also show H3K27ac signal enrichment at the rs55958994 locus in K562 (leukemia), HeLa (cervical cancer), HepG2 (liver cancer), MCF-7 (breast cancer), HCT-116 (colon cancer), and PANC-1 (pancreatic cancer) cells (fig. S2A). We found that both alleles, as in PCa cells, enhanced promoter activity in these cell lines, and a stronger effect was seen in the risk (T) allele than the nonrisk allele (C) (fig. S2B). These results suggest that the risk allele of rs55958994 may be associated with multiple types of cancer.

We next used the CRISPR-Cas9 technique to delete a 2.56-kb enhancer fragment in the KRT8 intron region that contains rs55958994 in 22Rv1 cells; 22Rv1 cells are a diploid cancer cell line and thus are a good model for the creation of homozygous deletions (fig. S3A). Three homozygous deletion cell clones (fig. S3B) were characterized for tumor initiation, growth, and invasive migration and for the percentage of CSCs (Fig. 1, C to E). The soft agar colony formation assay showed that tumor formation was greatly reduced in the enhancer deletion knockout (KO) cells; the average number of colonies from the KO cells was about half the number from the wild-type (WT) controls (Fig. 1C). Using a Transwell assay that examines invasive migration, we found that the number of colonies from the KO cells that could migrate through the membrane was only 25% of the control cells (Fig. 1D). In addition, fluorescence-activated cell sorting (FACS) analysis showed a significant decrease in the CD44^+^CD24^−^ cell population (these cells are considered to be CSCs; Fig. 1E). These results suggest that the enhancer containing the risk SNP rs55958994 plays a critical role in PCa development and progression.

### Deletion of rs55958994-associated enhancer causes widespread effects on the transcriptome

**Fig. 2:**
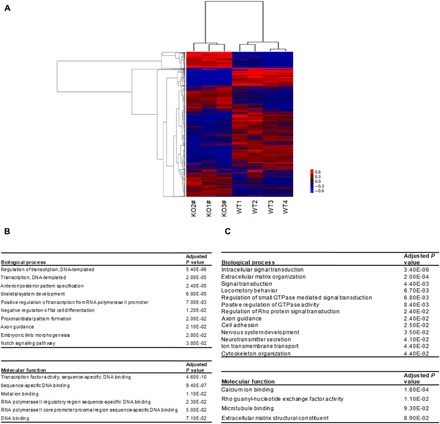
Transcriptomic changes following the deletion of the rs55958994-associated enhancer. (**A**) Comparison of gene expression in enhancer KO and WT cell lines. The heat map and dendrogram show clustering of differentially expressed genes in three KO and four WT 22Rv1 cell lines. (**B**) Annotation enrichment analysis of genes significantly down-regulated in KO compared to WT cells (fold change < 1 or 1.5 and adjusted *P* < 0.1). (**C**) Annotation enrichment analysis of genes significantly up-regulated in KO compared to WT conditions (fold change > 1.5 and adjusted *P* < 0.1). GTPase, guanosine triphosphatase.

To identify direct and indirect target genes regulated by the enhancer at the rs55958994 locus, we performed RNA-seq in WT control and KO cells (four and three biological replicates, respectively). The expression profiles of KO and WT samples are well segregated, as shown in the principal components analysis (PCA) and clustering results (fig. S3, C and D). RNA-seq analysis revealed global alterations of gene expression in the KO cells, with 2695 genes being significantly down-regulated (>1.5 folds; *P* < 0.1) in KO cells as compared to WT cells (Fig. 2A). Gene ontology analysis indicated that the differentially regulated genes are related to intracellular signaling, extracellular matrix organization, and cell migration (Fig. 2, B and C). These results are consistent with the phenotype of KO cells, which show reduced tumor formation and cell invasion. Moreover, genes that were down-regulated are candidates for downstream target genes of the rs55958994 risk enhancer in the complex regulatory network.

### The risk enhancer exerts long-range effects on gene regulation in PCa

**Fig. 3:**
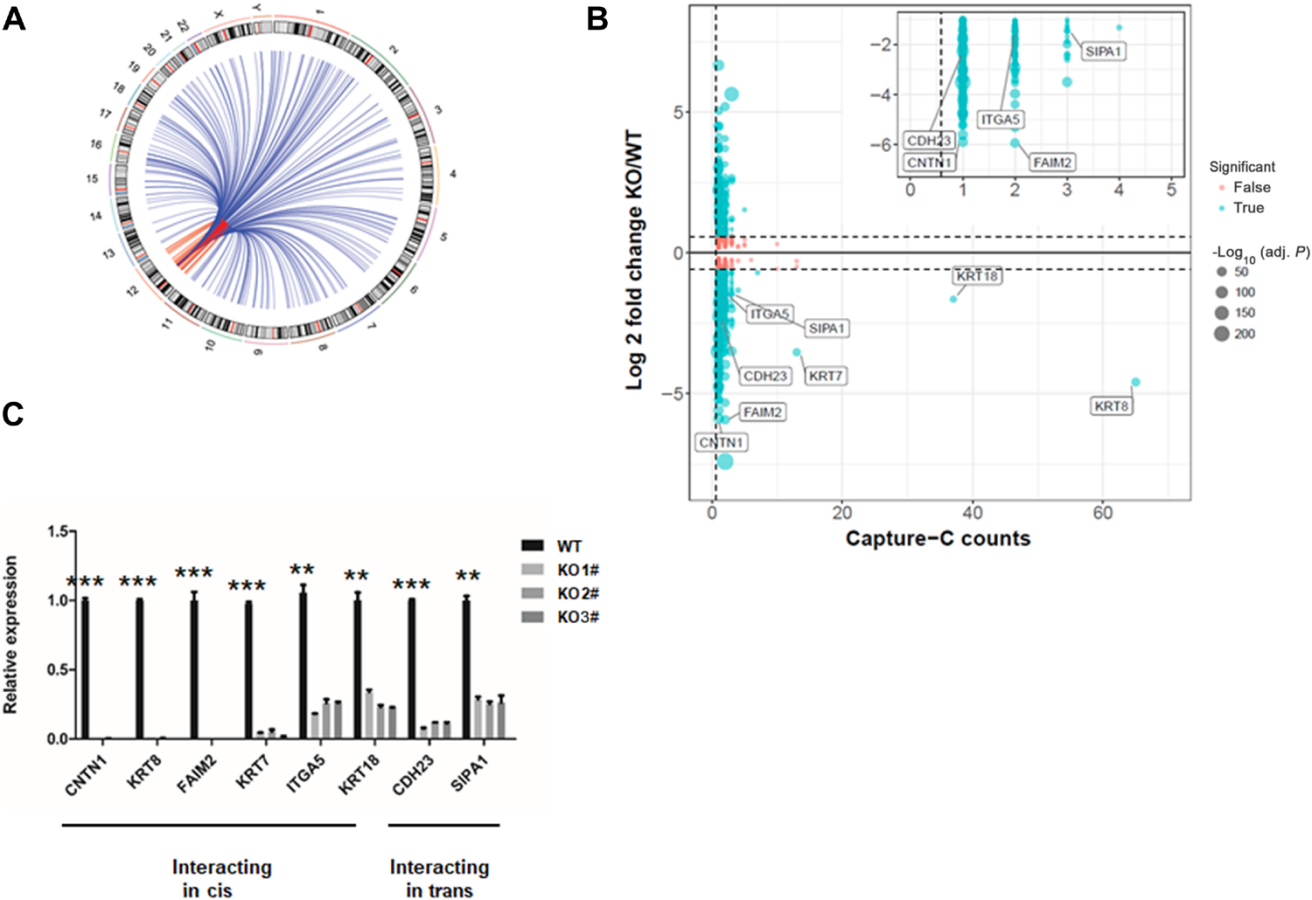
The rs55958994 risk enhancer is a hub for intrachromosomal and interchromosomal interactions. (**A**) Circos plot showing genome-wide interactions indicated by curves extending from the bait locus; intrachromosomal and interchromosomal interactions are in red and blue, respectively. Interactions reproducible in at least two biological replicates are shown. (**B**) A comparison of gene expression fold changes based on RNA-seq and the corresponding Capture-C signal counts are shown. A total of 2936 genomic loci with differential expression (adjusted *P* < 0.1) are shown on the plot. Among them, 219 loci with both reproducible interaction (Capture-C support > 1) and significant expression change between KO and WT (adjusted *P* < 0.1 and log 2 fold change > 1 or <−1) are shown in blue. (**C**) qRT-PCR analysis of mRNA (independent mRNA library) levels of potential target genes of the risk enhancer based on analysis in enhancer-deleted (KO) cells. Data represent means ± SEM of three independent experiments. ***P < 0.001, **P < 0.01 compared with corresponding KO cells.

We note that genes that were down-regulated are candidates for direct target genes of the rs55958994 risk enhancer. However, many of the down-regulated genes could also be indirect targets, affected as part of a network of regulation. To identify potential target genes that might be directly regulated by the rs55958994-associated enhancer through physical interactions, we performed Capture-C in PCa cells using the region that contains the rs55958994-associated enhancer as the Capture-C “bait.” We identified 658 long-range interacting sites that are reproducible in three biological replicates and 1845 long-range interacting sites that are reproducible in at least two biological replicates located in the same or different chromosomes (Fig. 3A). Among the 1845 doubly reproducible interacting sites, 1597 (86.56%) are located in cis while only 248 (13.44%) are in trans. Among the 658 triply reproducible interacting sites, 641 (97.42%) are located in cis while only 17 (2.58%) are in trans. To identify the genes that have physical contact with and are subject to transcriptional regulation by the risk enhancer, we combined the RNA-seq and Capture-C analyses (Fig. 3B). Among the genes having significant changes in transcript levels, eight represented genomic regions with high signals in the Capture-C assay (*KRT8*, *KRT18*, and *KRT7*; Fig. 3B) or positive Capture-C signal (*SIPA1*, *ITGA5*, *CDH23*, *FAIM2*, and *CNTN1*; Fig. 3B) and were previously linked to cancer progression; two (*CDH23* and *SIPA1*) of the eight genes are located in trans, while the other six (*CNTN1*, *KRT8*, *FAIM2*, *KRT7*, *ITGA5*, and *KRT18*) are located in cis. Quantitative real-time polymerase chain reaction (qRT-PCR) confirmed that the expression of these candidate genes was significantly reduced in the risk enhancer deletion mutant (Fig. 3C). These eight genes that have significantly decreased expression upon enhancer deletion and enrichment of Capture-C signals in control cells are strong candidates as direct target genes of the rs55958994-associated enhancer.

### Re-expression of direct target genes of the rs55958994-associated enhancer rescues cancer onset and progression

**Fig. 4:**
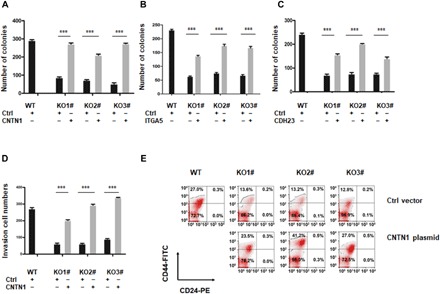
Rescue of tumorigenic phenotypes in enhancer-deleted 22Rv1 cells following the re-expression of candidate enhancer targets. Quantification of soft agar colony formation assays of WT 22Rv1 cells, three enhancer-deleted cell lines (KOs 1 to 3), and KO cells re-expressing CNTN1 (**A**), ITGA5 (**B**), or CDH23 (**C**). Data represent means ± SEM of three independent experiments. ***P < 0.001. (**D**) Quantification of Transwell assays of WT 22RVv1 cells, three enhancer-deleted cell lines (KOs 1 to 3), and KO cells re-expressing CNTN1. Data represent means ± SEM of three independent experiments. ***P < 0.001. (**E**) The percentage of CD44^+^CD24^−^ cells in WT 22Rv1 cells, three enhancer-deleted cell lines (KOs 1 to 3), and CNTN1 re-expressed KO cells.

Although the eight genes identified above may be directly regulated by the risk enhancer, it is unknown whether any of these genes are functional genes directly responsible for the defects in cancer phenotypes observed in the enhancer deletion mutant. To further determine whether these candidate genes are responsible for the tumorigenesis defects observed in the enhancer deletion mutant, we stably reintroduced these genes into the enhancer-deleted KO cells. Soft agar colony formation assays showed that re-expression of *CNTN1*, *ITGA5*, and *CDH23* could significantly rescue the decrease in colony numbers (Fig. 4, A to C, and fig. S4, A to C), while *KRT8* (fig. S5, A and B) and the other four tested genes could not. The three genes that could rescue the reduction in colonies in the soft agar assay are extracellular matrix proteins; ITGA5 is an integrin family member, CNTN1 is a neural-specific matrix protein, and CDH23 is a cadherin-related protein. Re-expression of *ITGA5*, *CDH23*, and *CNTN1* was verified by qRT-PCR (fig. S6, A to C) and Western blot (fig. S6, D to F). Although the rs55958994-associated enhancer is located in an intron of *KRT8* and KRT8 expression was reduced in the rs55958994-associated enhancer deletion cells, the expression of KRT8 did not rescue the defect of soft agar colony formation (fig. S5, A and B) and invasive migration ability (fig. S5, C and D) and the decrease in CSC population (fig. S5E). Re-expression of *KRT8* was verified by qRT-PCR (fig. S5F) and Western blot (fig. S5G). This suggests that, although *KRT8* is a direct target gene, it is not involved in the cancer risk associated with the risk enhancer. *CNTN1* and *ITGA5* are about 12 and 1.5 Mb away from the risk SNP on chromosome 12, whereas *CDH23* is on chromosome 10. Intrachromosome interactions between the rs55958994-associated enhancer and *CNTN1* or *ITGA5* and interchromosomal interactions between the enhancer and *CDH23* were verified by independent chromosome conformation capture (3C) assays (fig. S7, A to C). Furthermore, Transwell assays showed that re-expression of CNTN1 could rescue the invasive migration defect of the KO cells (Fig. 4D and fig. S4D) and result in an increase in the number of CD44^+^CD24^−^ cells (Fig. 4E). These results suggest that the risk-associated enhancer harboring rs55958994 may contribute, via long-range regulation of *CNTN1*, to the formation or maintenance of CSCs. No significant recovery of the invasive migration ability or the CSC population was found in KO cells that re-expressed ITGA5 and CDH23.

### SNP rs55958994 plays a critical role in function of the risk-associated enhancer

**Fig. 5:**
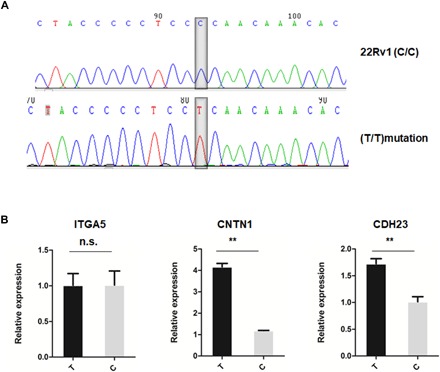
Risk allele of SNP rs55958994 in 22Rv1 cells affected the expression levels of CNTN1 and CDH23. (**A**) Site-directed mutation by CRSPR-Cas9 and sequencing of SNP rs55958994 nonrisk (C) and risk (T) alleles in 22Rv1cells. (**B**) *CDH23* and *CNTN1* expressions are increased in cells with risk (T) allele of rs55958994 compared with WT 22Rv1 cells with (C) allele of rs55958994, while no significant alteration of *ITGA5* expression was detected in cells with risk (T) allele of rs55958994. Data represent means ± SEM of three independent experiments. **P < 0.01. n.s., not significant.

As noted above, the deletion of the rs55958994-associated enhancer leads to the down-regulation of *ITGA5*, *CDH23*, and *CNTN1*, to defects of tumor initiation, growth, and invasive migration, and to a decrease in the percentage of CSCs. To examine whether the different alleles at rs55958994 had differential effects in these same assays, we used CRISPR-Cas9 to generate site-directed mutation of SNP rs55958994 in 22Rv1 PCa cells (C) to (T); Fig. 5A). The expression of *CDH23* and *CNTN1* is increased in cells with risk (T) allele of rs55958994, as compared to WT 22Rv1 cells that have the (C) allele of rs55958994 (Fig. 5B). However, we detected no significant change of expression levels of *ITGA5* in cells with the risk (T) allele of rs55958994. This demonstrates that SNP rs55958994 affects expression of some of the downstream target genes of the rs55958994-related enhancer.

**Fig. 6:**
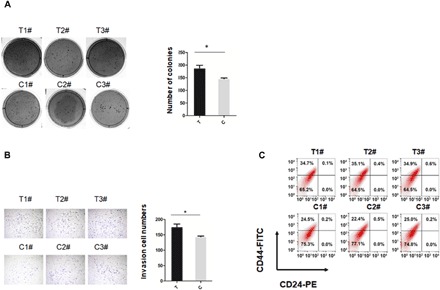
SNP rs55958994 is a functional SNP in 22Rv1 cells. (**A**) Soft agar colony formation assays comparing WT 22Rv1 cells with (C) allele of rs55958994 and 22Rv1 cells with (T) allele of rs55958994. Quantification is at right. Data represent means ± SEM of three independent experiments. *P < 0.05. (**B**) Transwell assays in WT 22Rv1 cells with (C) allele of rs55958994 and 22Rv1 cells with (T) allele of rs55958994. Cells that had migrated to lower chambers were stained with 0.1% crystal violet. Quantification is at right. Data represent means ± SEM of three independent experiments. *P < 0.05. (**C**) FACS analysis of the cell surface markers CD44 and CD24 in WT 22Rv1 cells with (C) allele of rs55958994 and in 22Rv1 cells with (T) allele of rs55958994.

The soft agar colony formation assay indicated that tumor formation was significantly increased in the 22Rv1 cells with the risk (T) allele of rs55958994, as compared with WT 22Rv1 cells with the (C) allele of rs55958994; the average number of colonies from the 22Rv1 cells with the risk (T) allele of rs55958994 was increased by 1.3-fold compared to the WT 22Rv1 cells with the risk (C) allele (Fig. 6A). Transwell assays showed that the number of colonies from the 22Rv1 cells with the risk (T) allele of rs55958994 that could migrate through the membrane was about 1.25-fold of the WT 22Rv1 cells with the risk (C) allele (Fig. 6B). FACS analysis showed a significant increase in the CD44^+^CD24^−^ cell population in the 22Rv1 cells with the risk (T) allele of rs55958994 compared with the WT 22Rv1 cells with the risk (C) allele (Fig. 6C). These results suggest that the risk SNP rs55958994 is an important functional component of the enhancer that influences PCa development and progression.

In addition, we performed RNA-seq in the 22Rv1 cells with the risk (T) allele of rs55958994 compared with the WT 22Rv1 cells with the risk (C) allele. We identified 2227 genes whose expression is down-regulated in the enhancer KO compared to WT cell lines; these 2227 genes represent genes whose expression is normally increased by the enhancer. We identified 798 genes that are up-regulated in cells containing the risk allele T, as compared to cells containing the nonrisk C allele (fold change > 1.5 and adjusted *P* < 0.1); these 798 genes represent genes whose expression is increased when the risk allele is present (including both direct and indirect targets). There are 314 overlapped genes in these two gene sets (fig. S8A). We identified 1170 genes whose expression is up-regulated in the enhancer KO compared to WT cell lines; these genes represent genes (most likely indirect targets) whose expression level is reduced by the presence of the enhancer. We also identified 238 genes that are down-regulated in cells containing the risk allele T versus cells containing the nonrisk C allele (fold change > 1.5 and adjusted *P* < 0.1); these 238 genes represent genes whose expression is decreased when the risk allele is present. There are 28 overlapped genes in these two gene sets (fig. S8B). Among the eight potential target genes described above, CNTN1, ITGA5, and CDH23 were up-regulated in the 22Rv1 cells with the risk (T) allele of rs55958994 as compared with the WT 22Rv1 cells with the risk (C) allele (fig. S8C). However, our qRT-PCR data showed no significant change in ITGA5 expression level in the 22Rv1 cells with risk (T) allele of rs55958994. This is consistent with the hypothesis that the risk allele may have a smaller effect than the enhancer deletion. These data suggest that SNP rs55958994 is associated with the function of the enhancer in regulating gene expression and cancer progression.

## DISCUSSION

3C technologies have enabled studies of the dynamics of high-order chromatin structures and long-range chromatin interactions (*26*–*30*). However, how these long-range interactions contribute to biological processes and human diseases is not well understood. Our research has focused on an enhancer containing the PCa risk–associated rs55958994 SNP, integrating higher-order chromatin structure data, gene expression profiling, and functional assays. Our results suggest that the rs55958994-containing enhancer regulates PCa progression through long-range interactions with multiple genes. Among these genes, *ITGA5*, *CDH23*, and *CNTN1* were verified to be both regulated by the rs55958994-containing enhancer and associated with PCa progression; deletion of the enhancer region in PCa cells induced down-regulation of these target genes and led to defects in tumor initiation and migration and loss of CSCs.

Previous eQTL (expression quantitative trait locus) analyses suggested that rs55958994 is linked to KRT8 expression level (*31*) and its high linkage disequilibrium (LD) variants to KRT8 and KRT18 expression levels (*32*, *33*). In our research, on the basis of 3C assays and RNA-seq of cells lacking the enhancer, we observed that the rs55958994-associated enhancer could regulate the expression of multiple genes, which include *KRT8*, *KRT7*, *KRT18*, *CNTN1*, *FAIM2*, *ITGA5*, *CDH23*, and *SIPA1*. Some of these genes are on the same chromosome as the enhancer (e.g., *KRT8*, *KRT18*, *ITGA5*, and *CNTN1*), and other genes are on a different chromosome (e.g., *CDH23*). Previous studies have reported the possibility that risk SNPs may regulate gene expression through long-range intrachromatin interactions in cancer cells. For example, PCa risk SNP rs1859962 is within a 130-kb LD block that lies in a gene desert at the 17q24.3 locus. A PCa-specific enhancer in this LD block may regulate the *SOX9* oncogene by establishing a 1-Mb chromatin loop (*34*). Luo *et al.* (*16*) found that a PCa risk region located in the second intron of the *JAZF1* gene inhibits the expression of *HOXA13*, which is about 870 kb away from the regulatory element. We show here that the rs55958994-associated enhancer may regulate PCa progression not only through long-range intrachromosomal interactions but also through the interchromosomal regulation of several target genes. Szabo and colleagues (*35*) have previously shown trans inter-TAD (topologically associating domain) contact events in the *Drosophila* genome. In addition, Miao *et al.* (*36*) and Patel *et al.* (*37*) have reported interchromosomal interactions. However, the involvement of interchromosomal interactions in enhancer-mediated gene regulation in human cells has not been well studied. Although our work using Capture-C and independent 3C assays suggests the possibility of interchromosomal regulation of *CDH23* by the rs55958994-associated enhancer, further studies are needed to verify this discovery. Our study showed that mutation of SNP induced alteration of target genes’ expression levels (Fig. 3C). The alteration of enhancer activity may be associated with change of transcription factors binding to the SNP. Future studies will identify the transcription factor that might differentially bind to the risk alleles. Although the deletion of the rs55958994-associated risk enhancer causes significant down-regulation of its target gene *ITGA5*, mutation of rs55958994 did not cause notable expression change of this gene (Fig. 5B). It is possible that the change in ITGA5 expression in rs55958994 mutant cells is not significant enough to be detected and that a different SNP may have a stronger effect on ITGA5 expression.

We demonstrate that re-expression of both intrachromosomal and interchromosomal target genes of the rs55958994-associated enhancer could rescue some of the phenotypic defects observed in the enhancer KO cells. For example, re-expression of *CNTN1*, *ITGA5*, and *CDH23* increased the numbers of colonies in the enhancer KO cells, affecting important properties of cancer cell growth. Thus, the regulation of PCa progression by the rs55958994-associated enhancer may involve a complex network of multiple target genes. Previous studies have reported that *KRT8* (*38*) and *ITGA5* (*39*) play important roles in cancer progression. ITGA5 is a member of the integrin family, and Xiao *et al.* (*40*) reported that ITGA5 regulates triple-negative breast cancer stemness and metastasis through the Src/Vav2/Rac1 pathway. In addition, *KRT18* (*41*), *CNTN1* (*42*), and *SIPA1* (*43*) have previously been reported as related to PCa. Although re-expression of chromatin-interacting target genes of rs55958994-associated enhancer such as KRT8, FAIM2, KRT18, and SIPA1 in enhancer-deleted PCa cells did not result in a significant increase of cell growth from our experiments, we cannot exclude their involvement in PCa; it is possible that these genes play roles in PCa progression that are not measured in the assays used in our studies.

CSCs or cancer-initiating cells are a subpopulation of cancer cells that are capable of initiating new tumors. Targeting of CSCs is an extremely important strategy in cancer clinical therapy because of their ability to induce drug resistance and to form metastases (*3*, *4*). CSCs have been identified in several different kinds of tumors, including PCa (*44*). Our data show that disruption of the rs55958994-associated enhancer decreases the population of CSC.We show that re-expression of *CNTN1* (a direct target gene of the risk enhancer that is linked to the enhancer by long-range intrachromosomal interactions) can rescue the defects in the number of CSCs. Previous research has shown that CNTN1 functions as a neural cell adhesion protein and plays various roles in neural development (*45*). Recently, Yan and colleagues (*42*) also reported that *CNTN1* overexpression induced elevated AKT activation and reduced E-cadherin (CDH1) in DU145 cells and corresponding xenograft tumors and that knockdown of *CNTN1* reduced tumor initiation mediated by PCa stem-like cells, which is consistent with the results in our presented research. Our data suggest that potential clinical intervention to reduce tumor phenotypes mediated by the rs55958994-associated enhancer could be to target *CNTN1* and/or to use other methods to reduce PCa stem cells. In addition, recent research has demonstrated that long-range chromatin interactions can be disrupted through a special CRISPR system or artificial zinc fingers (*40*, *46*, *47*). Perhaps, modifying the long-range interaction network of the rs55958994-associated enhancer could pose as an alternative way of developing a previously unidentified PCa treatment method.

In summary, we conclude that the region including risk SNP rs55958994 at 12q13 is a functional component of an enhancer that plays an important role in PCa development and progression, by regulating a complex gene network via long-range intrachromatin and interchromatin interactions.

## MATERIALS AND METHODS

### Cell culture

The human PCa cell line 22Rv1, C4-2B, and LNCaP cells were obtained from the American Type Culture Collection (ATCC). 22Rv1, C4-2B, and LNCaP cells were incubated at 37°C with 5% CO_2_ and cultured in complete RPMI 1640 Medium modified (HyClone), supplemented with 10% fetal bovine serum (FBS) (Biological Industries) and 1% penicillin/streptomycin (Gibco). The human cervical cancer cell line HeLa, liver cancer cell line HepG2, and breast cancer cell line MCF-7 were obtained from the ATCC, incubated at 37°C with 5% CO_2_, and cultured in Dulbecco’s modified Eagle’s medium (HyClone), supplemented with 10% FBS (Biological Industries) and 1% penicillin/streptomycin (Gibco).

**Chromatin immunoprecipitation sequencing**

ChIP-seq assay was performed, as previously described with modification (*48*). Briefly, 1 × 10^7^ cells were cross-linked with 1% formaldehyde and harvested in Farnham lysis buffer [50 mM (pH 7.5) tris-HCl, 150 mM NaCl, 5 mM EDTA, 0.5% NP-40, and 1% Triton X-100] supplemented with 1 mM phenylmethylsulfonyl fluoride and 1× protease inhibitor cocktail. Sonication was carried out to shear the chromatin to yield DNA fragment sizes of 100 to 500 base pairs (bp). Samples were cleared by centrifuging at 12,000*g* for 10 min at 4°C and precleared for 1 hour with 40 μl of protein A/G agarose beads (no. 20421, Thermo Fisher Scientific). Part of the precleared samples was used as input, and the remainder was immunoprecipitated with 5 μg of H3K27ac antibody (ab4729, Abcam) and 60 μl of protein A/G agarose beads. The precipitated DNA and input DNA were sequenced using an Illumina HiSeq 4000 sequencer. Reads were mapped to the GRCh37/hg19 reference genome using HISAT2 (*49*) and further analyzed with HOMER (Hypergeometric Optimization of Motif EnRichment) package (*50*).

### Luciferase reporter assays

The H3K27ac region containing rs55958994 (chr12: 53300245 to 53303204, UCSC.hg19) was amplified from 22Rv1 genomic DNA and cloned into the pGL3-Promoter vector (E1761, Promega) upstream of SV40 promoter; the region for analysis was selected on the basis of the H3K27ac ChIP-seq data from 22Rv1 cells. The introduction of the T versus C allele at rs55958994 was obtained by site-directed mutagenesis. Reporter plasmids and the pRL-TK *Renilla* luciferase control vector (E2241, Promega) were cotransfected into 22Rv1, C4-2B, or LNCaP cells using Lipofectamine 3000 reagent (Invitrogen). Cells were harvested for luciferase assays, and luciferase activity was determined using the Dual-Luciferase Reporter Assay System (E1910, Promega); the luminescent signals from experimental samples were normalized to controls.

### Soft agar assays

Culture medium (1.5 ml) containing 0.6% NuSieve GTG agarose (no. 50081, Lonza) was plated into six-well plates and allowed to solidify at 4°C. A final concentration of 0.3% agarose containing 5000 22Rv1 cells was carefully plated on top of the solidified GTG agarose. Plates were incubated at 37°C with 5% circulating CO_2_, and the medium was changed every 3 days. After 2 to 3 weeks, colonies were stained with 0.005% crystal violet in 4% paraformaldehyde solution, and the number of colonies was counted.

### Transwell invasion assays

The upper chambers of the Transwell plates (8 μm; Corning Costar) were precoated with diluted matrigel (BD Biosciences). 22Rv1 cells were seeded into the upper chambers (1 × 10^5^ per chamber) with 200 μl of serum-free RPMI 1640 Medium modified, and 500 μl of the complete RPMI 1640 Medium modified was added into the lower chambers. After 48 hours of incubation, cells that were migrating to lower chambers were dyed with 0.1% crystal violet and counted under the microscope.

### Flow cytometry analysis

22Rv1 cells were digested with 0.25% trypsin, washed with cold Hanks’ balanced salt solution (HBSS) once, followed by resuspending in 90 μl of cold HBSS, and then stained with 5 μl of anti–CD44-FITC (fluorescein isothiocyanate; eBioscience) and 5 μl of anti–CD24-PE (phycoerythrin; eBioscience) in the dark for 25 min on ice. Flow cytometry analysis was performed using the BD Accuri C6 Flow Cytometer (BD Biosciences).

### RNA sequencing

Barcoded RNA-seq libraries were sequenced as 150-bp paired-end reads using the Illumina HiSeq 4000 platform. Reads were mapped to the reference genome (GRCh37/hg19) using TopHat2 (*51*) with a GENCODE GTF file supplied as gene model annotations. HTSeq (*52*) was used to quantitate the abundance of transcripts for each gene. DESeq2 (*53*) was used to perform normalization and regularized log transformations on read counts. PCA was conducted with regularized log-transformed data. Hierarchical clustering was performed over samples with replicates. Euclidean distance and complete linkage were used as a clustering metric and method, respectively. Lists of genes with differential transcript abundance between enhancer KO and WT samples were obtained on the basis of the following criteria: (i) Benjamini-Hochberg adjusted *P* < 0.1 and (ii) fold change of transcript abundance (enhancer KO versus WT) >1.5 for up-regulation or <1 or 1.5 for down-regulation. Differentially expressed genes were subjected to functional classification analysis with DAVID (Database for Annotation, Visualization and Integrated Discovery) version 6.8 (*54*, *55*). We applied Gene Cluster 3.0 (http://bonsai.hgc.jp/~mdehoon/software/cluster/software.htm) software to perform clustering analysis on the gene expression data. Genes were centered by means across samples, and hierarchical clustering was performed over genes and samples. The similarity metric of Euclidean distance and the clustering method of complete linkage were used. Clustering results were examined and visualized in Java TreeView (http://jtreeview.sourceforge.net/).

### Capture chromosome conformation capture

Capture-C was performed, as previously described (*30*). Two biotinylated DNA oligonucleotides were designed for both ends of the region containing the rs55958994 SNP, with the following sequence.

(5′ to 3′):

Biotin-gatctaggcagcacaaggagcaaaaaaacagcaccccaaagcctttcctgaaattccagtcccctccaccccacctttctggttccct.

5′-Biotin-ttcagagaaggccctgagaccctgagcagtggcattaagtcttggggccagtggggtggggaggtagctgttatcatcactcatgatc-3′.

22Rv1 cells (1 × 10^7^) were used to generate a standard 3C library with DpnII digestion. The purified 3C library DNA was then sheared to 200- to 300-bp fragments using sonication. The precapture DNA libraries were prepared using the NEBNext DNA Library Kit according to the manufacturer’s instructions (E6040, E7335, and E7500; New England BioLabs). The biotinylated probes and streptavidin beads (Invitrogen) were used to enrich the bait and its linked chromatin loci by two rounds of hybridization-capture approach. The barcoded Capture-C libraries were sequenced as 150-bp paired-end reads using the Illumina HiSeq 4000 platform. Capture-C data were analyzed on the basis of pipeline proposed by Davies *et al.* (*30*).

### CRISPR-Cas9–mediated deletions and mutation

To delete the SNP rs55958994-containing enhancer-like region, 22Rv1 cells were transfected with plasmids containing Cas9 and guide RNAs targeting the enhancer-like region. Colonies were derived from single cells and tested for the loss of the target region. Cell clones were genotyped, and three clones with homozygous deletion of the rs55958994-containing region and three control clones were used for RNA-seq analysis. The single-guide RNA (sgRNA) information is listed in table S1, which was designed by using web-based tools at https://zlab.bio/guide-design-resources. To generate site-directed mutation of SNP rs55958994, we transfected 22Rv1 PCa cells with plasmids containing Cas9, guide RNAs targeting the enhancer-like region, and DNA fragments containing (T) allele as repair templates. Double-nicking strategy was taken to reduce undesirable off-target mutagenesis. The sgRNA information is listed in table S4, which was designed by using web-based tools at https://zlab.bio/guide-design-resources. The region containing mutation was amplified using specific primers (table S4), and SNP genotyping was performed by Sanger sequencing–based method.

### RNA isolation and qRT-PCR assays

Total RNA was isolated from cultured cells using an RNeasy Mini kit (no. 74104, Qiagen) according to the manufacturer’s instructions. Complementary DNAs (cDNAs) were synthesized from 2 μg of total RNA using a PrimeScript RT reagent kit with a gDNA Eraser kit (no. RR047B, Takara). qRT-PCR was performed with FastStart Universal SYBR Green Master (Roche) in the Bio-Rad iQ5 System. The relative expression of target genes was determined using the comparative CT (cycle threshold) method and normalized with housekeeping gene HPRT1. Primer information is listed in table S2.

### Re-expression of target genes

Candidate target genes’ coding sequence (CDS) was cloned in pcDNA3.1 (flag tag)/Puro. Control plasmid and the plasmid with the target genes’ CDS clones were transfected into the enhancer KO 22Rv1 cells. The cells were treated with puromycin to select the cells that stably expressed the target genes. qRT-PCR and Western blot were used to verify the stable expression of the target genes.

### 3C assays

22Rv1 cells (1 × 10^7^) were used to prepare 3C libraries using HindIII digestion, according to the previously described method (*29*). The interactions between the SNP rs55958994 gene locus and the candidate partners were detected with PCR experiment. Primer information is listed in table S3.

### Western blots

Proteins were collected from cells using radioimmunoprecipitation assay buffer supplemented with 1× protease inhibitor cocktail. Sample loading was based on the results of bicinchoninic acid assay. Proteins were separated by SDS–polyacrylamide gel electrophoresis and then transferred to polyvinylidene difluoride membranes (0.45 μm; Millipore, Bedford, MA). The membranes were blocked and incubated with primary antibody overnight at 4°C, followed by secondary antibodies for 1 hour at room temperature. The blot bands were detected by ImageQuant LAS 4000 with an enhanced chemiluminescence kit (Thermo Scientific Pierce, Waltham, MA).

### Statistical analysis

Data represent means ± SEM. Statistical analysis was performed using Student’s *t* test (**P < 0.01 and ***P < 0.001). Statistical tests and plotting in Figs. 2 and 3 (A and B) were performed using R (www.r-project.org/).

## Supplementary Material

http://advances.sciencemag.org/cgi/content/full/5/7/eaaw6710/DC1

Download PDF

## SUPPLEMENTARY MATERIALS

Supplementary material for this article is available at http://advances.sciencemag.org/cgi/content/full/5/7/eaaw6710/DC1 Fig. S1. Fine-mapped PCa risk SNPs in 12q13 region. Fig. S2. SNP rs55958994 is associated with enhancer activity in cervical, liver, and breast cancer cell lines. Fig. S3. CRISPR-Cas9–mediated deletion of the rs55958994-associated enhancer. Fig. S4. Re-expression of target genes in enhancer-deleted PCa cells promotes cancer initiation, growth, and progression. Fig. S5. Re-expression of *KRT8* in enhancer-deleted PCa cells did not rescue the defect of soft agar colony formation and invasive migration ability and the decrease in CSC population. Fig. S6. Validation of target gene expression in enhancer KO cells. Fig. S7. 3C-PCR analysis confirms interaction of the rs55958994-associated enhancer and target gene loci. Fig. S8. Comparison of transcriptomic changes following deletion of the rs55958994-associated enhancer and mutation of rs55958994. Table S1. Guide RNAs for CRISPR-Cas9–mediated deletions. Table S2. Primers used in qRT-PCR experiment. Table S3. Primers for 3C-PCR assay. Table S4. Guide RNAs for CRISPR-Cas9–mediated SNP editing.
