# Supplementary material for: The prostate cancer risk variant rs55958994 regulates multiple gene expression through extreme long-range chromatin interaction to control tumor progression
Source: Sci Adv. 2019 Jul 17;5(7):eaaw6710. doi: 10.1126/sciadv.aaw6710 (PMC6636982; doi:10.1126/sciadv.aaw6710)
Supplement: http://advances.sciencemag.org/cgi/content/full/5/7/eaaw6710/DC1 [file supp_5_7_eaaw6710__index.html]

Science Advances | Science AdvancesAAASSearchScience AdvancesMenu

## Supplementary Materials

**This PDF file includes:**

- Fig. S1. Fine-mapped PCa risk SNPs in 12q13 region.
- Fig. S2. SNP rs55958994 is associated with enhancer activity in cervical, liver, and breast cancer cell lines.
- Fig. S3. CRISPR-Cas9–mediated deletion of the rs55958994-associated enhancer.
- Fig. S4. Re-expression of target genes in enhancer-deleted PCa cells promotes cancer initiation, growth, and progression.
- Fig. S5. Re-expression of *KRT8* in enhancer-deleted PCa cells did not rescue the defect of soft agar colony formation and invasive migration ability and the decrease in CSC population.
- Fig. S6. Validation of target gene expression in enhancer KO cells.
- Fig. S7. 3C-PCR analysis confirms interaction of the rs55958994-associated enhancer and target gene loci.
- Fig. S8. Comparison of transcriptomic changes following deletion of the rs55958994-associated enhancer and mutation of rs55958994.
- Table S1. Guide RNAs for CRISPR-Cas9–mediated deletions.
- Table S2. Primers used in qRT-PCR experiment.
- Table S3. Primers for 3C-PCR assay.
- Table S4. Guide RNAs for CRISPR-Cas9–mediated SNP editing.

Download PDF

**Files in this Data Supplement:**

- Adobe PDF - aaw6710\_SM.pdf
