# Supplementary material for: The prostate cancer risk variant rs55958994 regulates multiple gene expression through extreme long-range chromatin interaction to control tumor progression
Source: Sci Adv. 2019 Jul 17;5(7):eaaw6710. doi: 10.1126/sciadv.aaw6710 (PMC6636982; doi:10.1126/sciadv.aaw6710)
Supplement: Download PDF [file aaw6710_SM.pdf]

Science Advances
AAAS

advances.sciencemag.org/cgi/content/full/5/7/eaaw6710/DC1

Supplementary Materials for

The prostate cancer risk variant rs55958994 regulates multiple gene expression through extreme long-range chromatin interaction to control tumor progression

Yuyang Qian, Lei Zhang, Mingyang Cai, Hongxia Li, Heming Xu, Hongzhen Yang, Zhongfang Zhao, Suhn Kyong Rhie, Peggy J. Farnham, Jiandang Shi*, Wange Lu*

*Corresponding author. Email: wangelu@usc.edu (W.L.); shijd@nankai.edu.cn (J.S.)

Published 17 July 2019, *Sci. Adv.* **5**, eaaw6710 (2019)
DOI: 10.1126/sciadv.aaw6710

This PDF file includes:

Fig. S1. Fine-mapped PCa risk SNPs in 12q13 region.
Fig. S2. SNP rs55958994 is associated with enhancer activity in cervical, liver, and breast cancer cell lines.
Fig. S3. CRISPR-Cas9–mediated deletion of the rs55958994-associated enhancer.
Fig. S4. Re-expression of target genes in enhancer-deleted PCa cells promotes cancer initiation, growth, and progression.
Fig. S5. Re-expression of *KRT8* in enhancer-deleted PCa cells did not rescue the defect of soft agar colony formation and invasive migration ability and the decrease in CSC population.
Fig. S6. Validation of target gene expression in enhancer KO cells.
Fig. S7. 3C-PCR analysis confirms interaction of the rs55958994-associated enhancer and target gene loci.
Fig. S8. Comparison of transcriptomic changes following deletion of the rs55958994-associated enhancer and mutation of rs55958994.
Table S1. Guide RNAs for CRISPR-Cas9–mediated deletions.
Table S2. Primers used in qRT-PCR experiment.
Table S3. Primers for 3C-PCR assay.
Table S4. Guide RNAs for CRISPR-Cas9–mediated SNP editing.

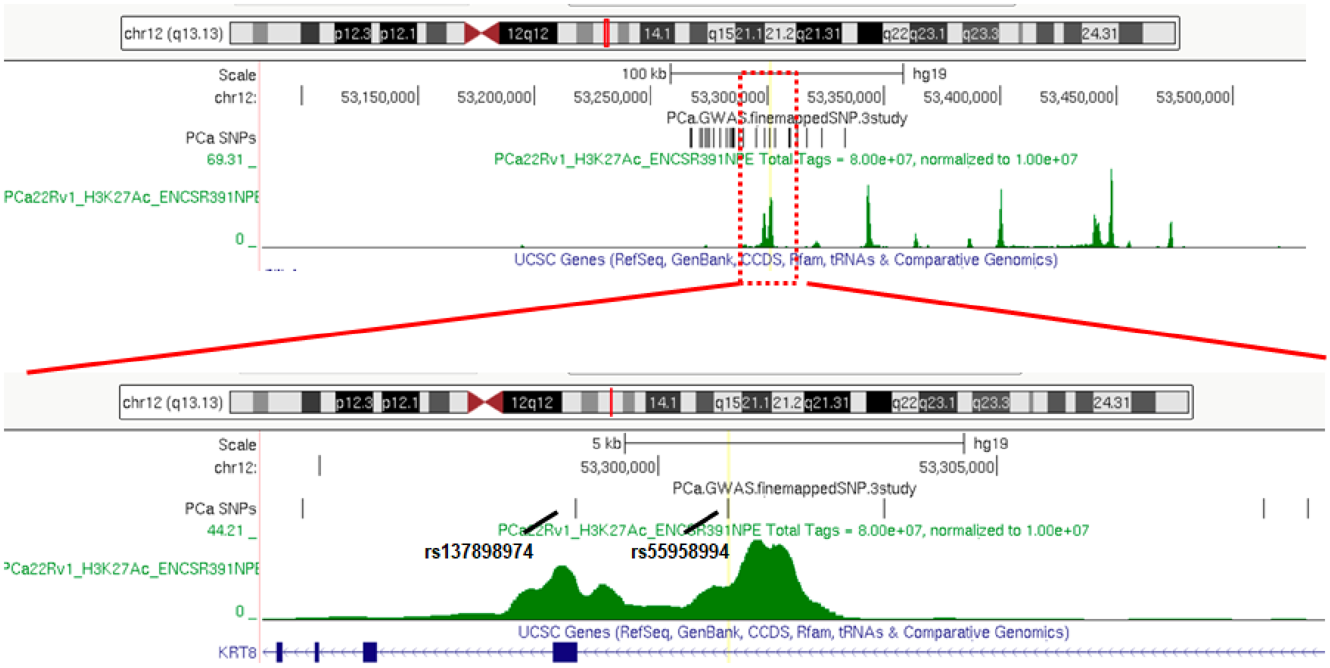

Fig. S1. Fine-mapped PCa risk SNPs in 12q13 region.

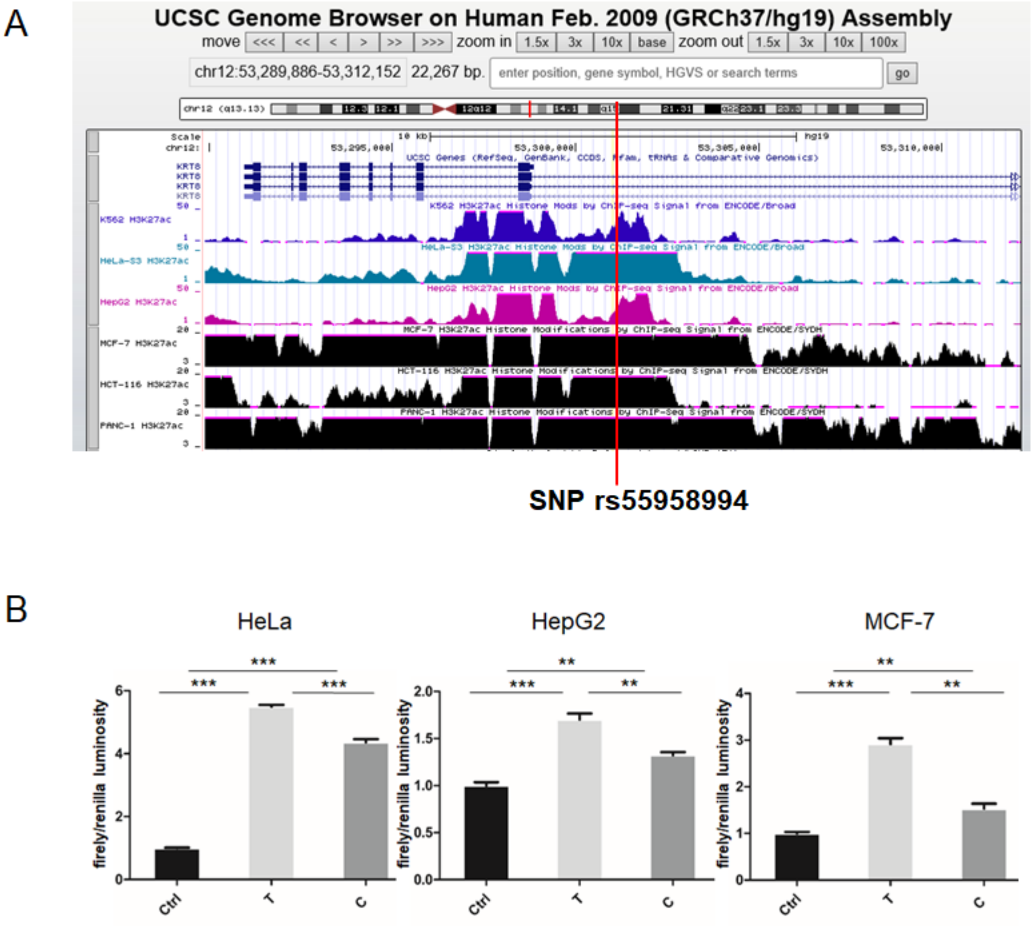

Fig. S2. SNP rs55958994 is associated with enhancer activity in cervical, liver, and breast cancer cell lines. (A) H3K27me3 ChIP data of the rs55958994-associated enhancer region in K562 (leukimia), Hela (cervical cancer), HepG2 (liver cancer), MCF-7 (breast cancer), HCT-116 (colon cancer) and PANC-1(pancreatic cancer) cells. (B) Luciferase reporter activity in Hela cells, HepG2 cells and MCF-7 cells transfected with luciferase reporters. Ctrl: luciferase reporter without the inserted enhancer; C: luciferase reporter with the rs55958994-associated enhancer region with the non-risk allele “(C)”; T: luciferese reporter with the rs55958994-associated enhancer region with the risk allele (T). Data represents means ± S.E.M. of three independent experiments; $**p < 0.01$, $***p < 0.001$

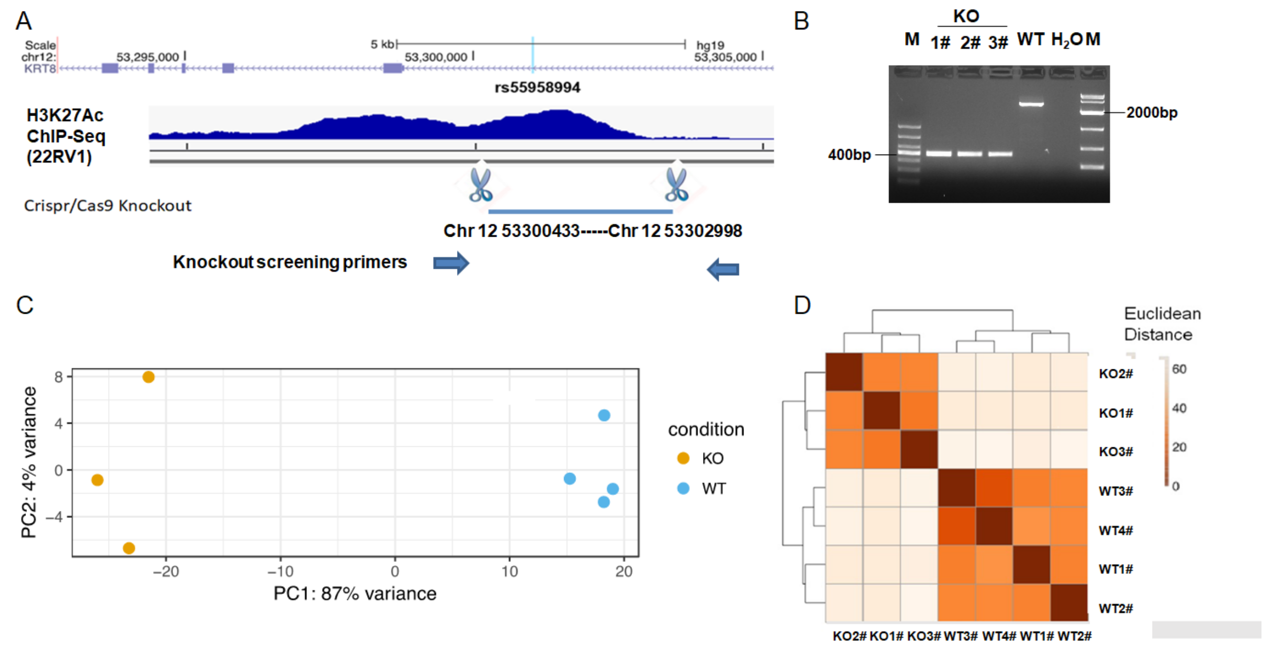

Fig. S3. CRISPR-Cas9–mediated deletion of the rs55958994-associated enhancer. (A) Schematic showing the region surrounding the rs55958994-associated enhancer region to be deleted and the knockout screening primers. (B) PCR analysis used to identify KO cell clones. A 400bp of PCR product indicates deletion of the rs55958994-associated enhancer region. PCR product of the intact enhancer region was shown in the WT lane. M: marker; H_2O Negative control, water as the PCR template; (C) Principal component analysis of RNA-seq results. Triplicates of KO samples and quadruplicates of WT samples were analyzed. (D) Clustered heatmap showing sample-to-sample similarity between RNA-seq profiles. Complete hierarchical clustering was performed based on the Euclidean distance between samples.

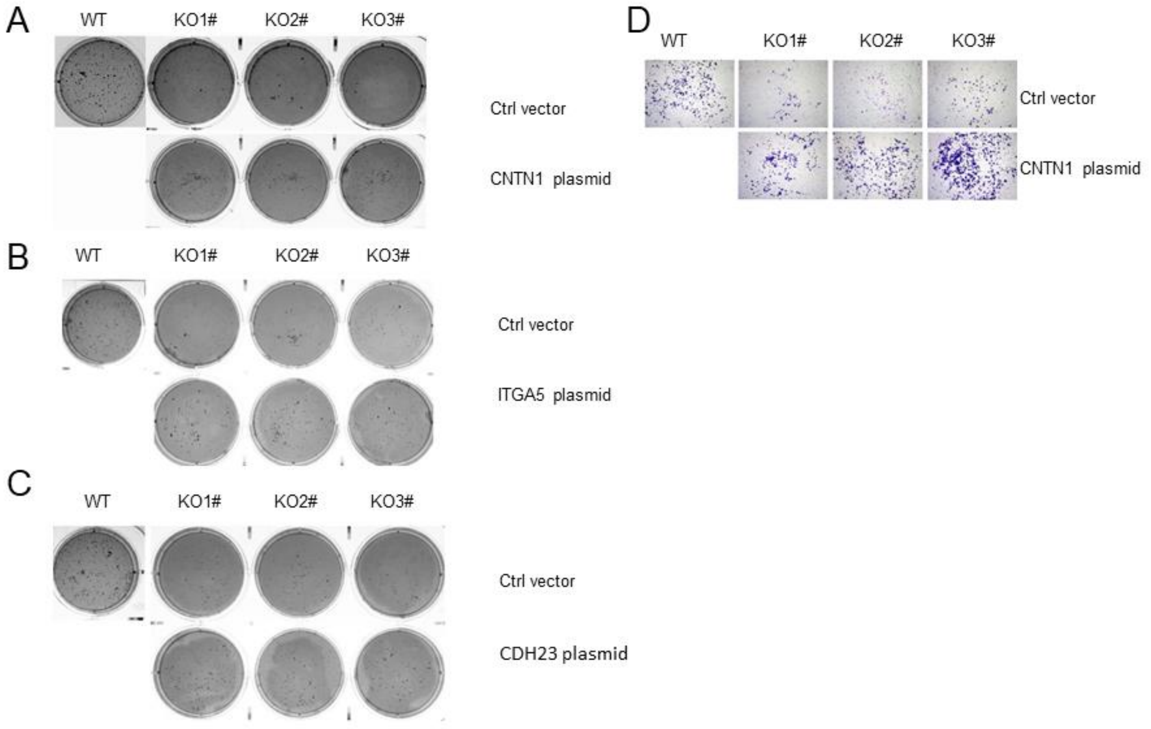

Fig. S4. Re-expression of target genes in enhancer-deleted PCa cells promotes cancer initiation, growth, and progression. Soft agar colony formation assays of wild-type 22Rv1 cells, 3 enhancer-deleted lines (KO 1-3) and KO cells re-expressing *CNTN1* (A), *ITGA5* (B) or *CDH23* (C). Quantification data are in Fig 4A-C. (D) Transwell assays of wild-type 22Rv1 cells, 3 enhancer-deleted lines (KO 1-3) and KO cells re-expressing *CNTN1*. Cells that had migrated to lower chambers were stained with 0.1% crystal violet. Quantification data are in Fig 4D.

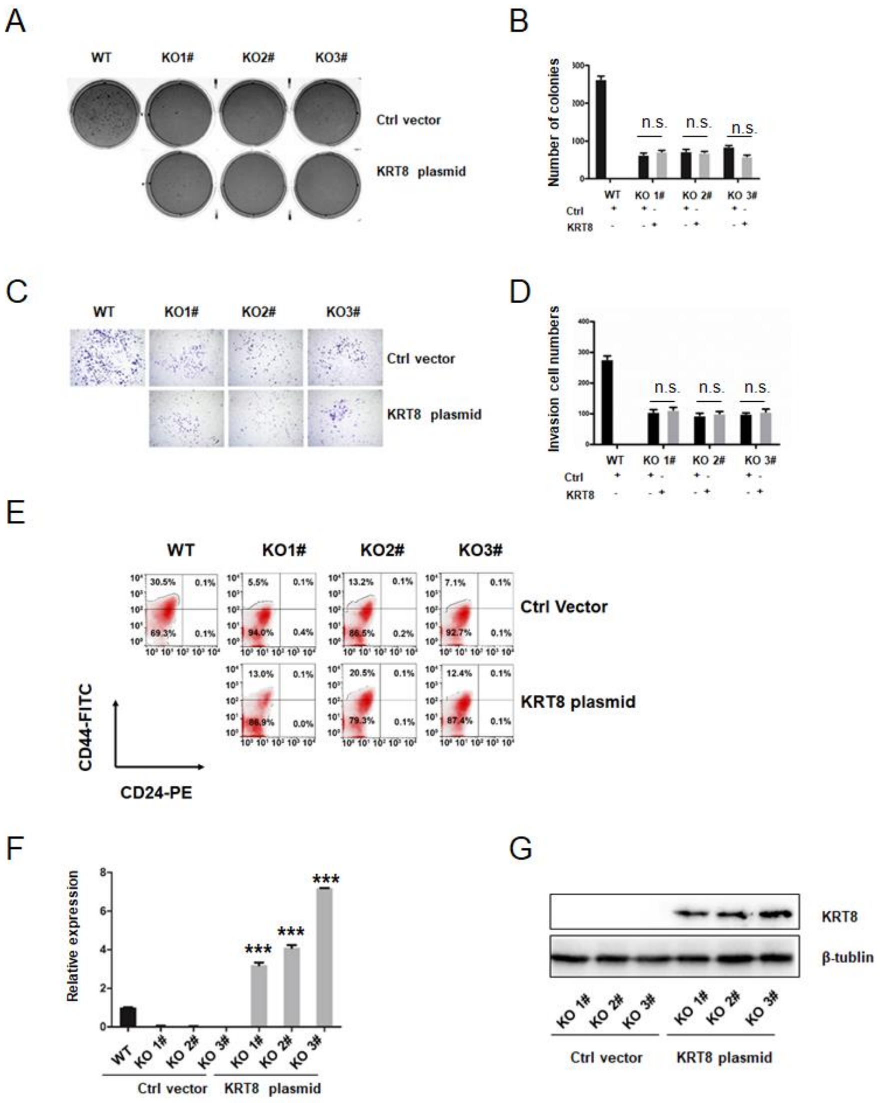

Fig. S5. Re-expression of *KRT8* in enhancer-deleted PCa cells did not rescue the defect of soft agar colony formation and invasive migration ability and the decrease in CSC population. (A) Soft agar colony formation assays of wild-type 22Rv1 cells, 3 enhancer-deleted lines (KO 1-3) and KO cells re-expressing *KRT8.* (B) Quantification data of Soft agar colony formation assays in (A). Data represents means ± S.E.M. of three independent experiments. (C) Transwell assays of wild-type 22Rv1 cells, 3 enhancer-deleted lines (KO 1-3) and KO cells re-expressing *KRT8*. Cells that had migrated to lower chambers were stained with 0.1% crystal violet. (D) Quantification data of transwell assays in (C). Data represents means ± S.E.M. of three independent experiments. ***p<0.001 compared with corresponding KO cells that were transfected with Ctrl vectors. (E) The percentage of CD44+CD24- cells in wild-type 22Rv1 cells, 3 enhancer-deleted lines (KO 1-3) and KRT8 re-expressed KO cells. (F) RT-qPCR analysis of *KRT8* mRNA in wild-type 22Rv1 cells, 3 enhancer-deleted lines (KO 1-3) and KO cells re-expressing *KRT8*. Data represents means ± S.E.M. of three independent experiments. ***p<0.001 compared with corresponding KO cells that were transfected with Ctrl vectors. (G) Western blot analysis of KRT8 in 3 enhancer-deleted lines (KO 1-3) and KO cells re-expressing *KRT8*. Data represents means ± S.E.M. of three independent experiments.

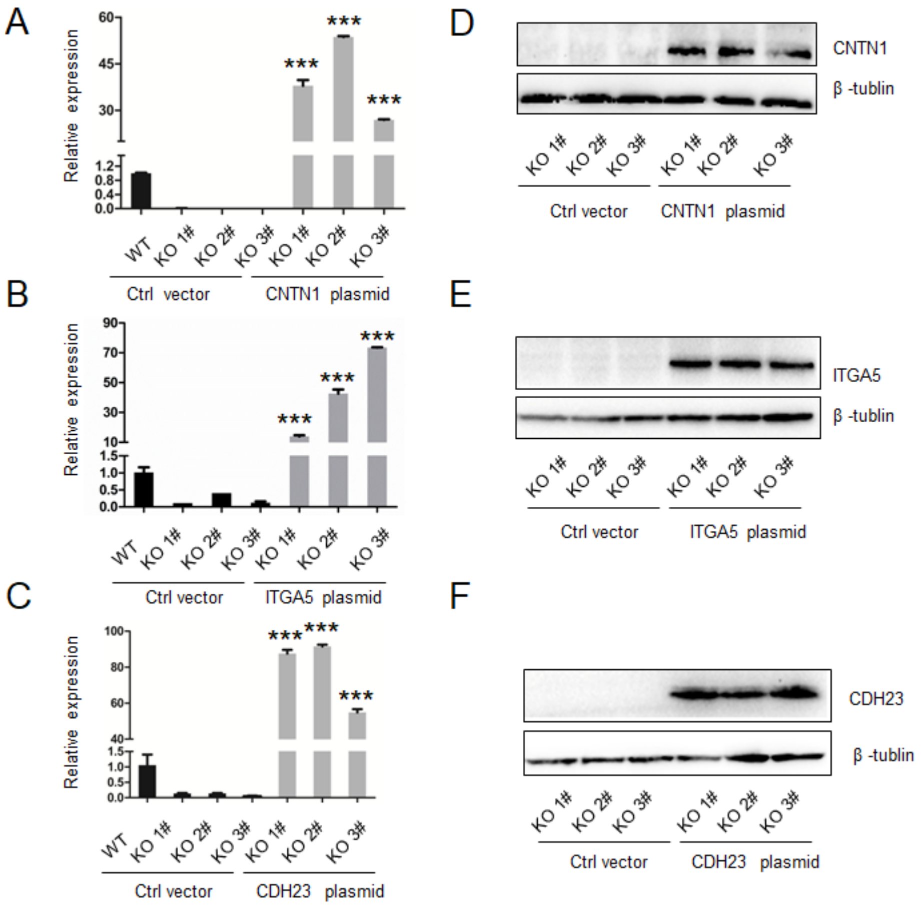

Fig. S6. Validation of target gene expression in enhancer KO cells. RT-qPCR analysis of *CNTN1* (A), *ITGA5* (B), and *CDH23* (C) mRNA in wild-type 22Rv1 cells, 3 enhancer-deleted lines (KO 1-3) and KO cells re-expressing *CNTN1*, *ITGA5*, or *CDH23*. Data represents means ± S.E.M. of three independent experiments. ***p<0.001 compared with corresponding KO cells that were transfected with Ctrl vector. Western blot analysis of CNTN1 **(D)**, ITGA5 **(E)**, and CDH23 **(F)** in 3 enhancer-deleted lines (KO 1-3) and KO cells re-expressing *CNTN1*, *ITGA5*, and *CDH23*. Data represents means ± S.E.M. of three independent experiments.

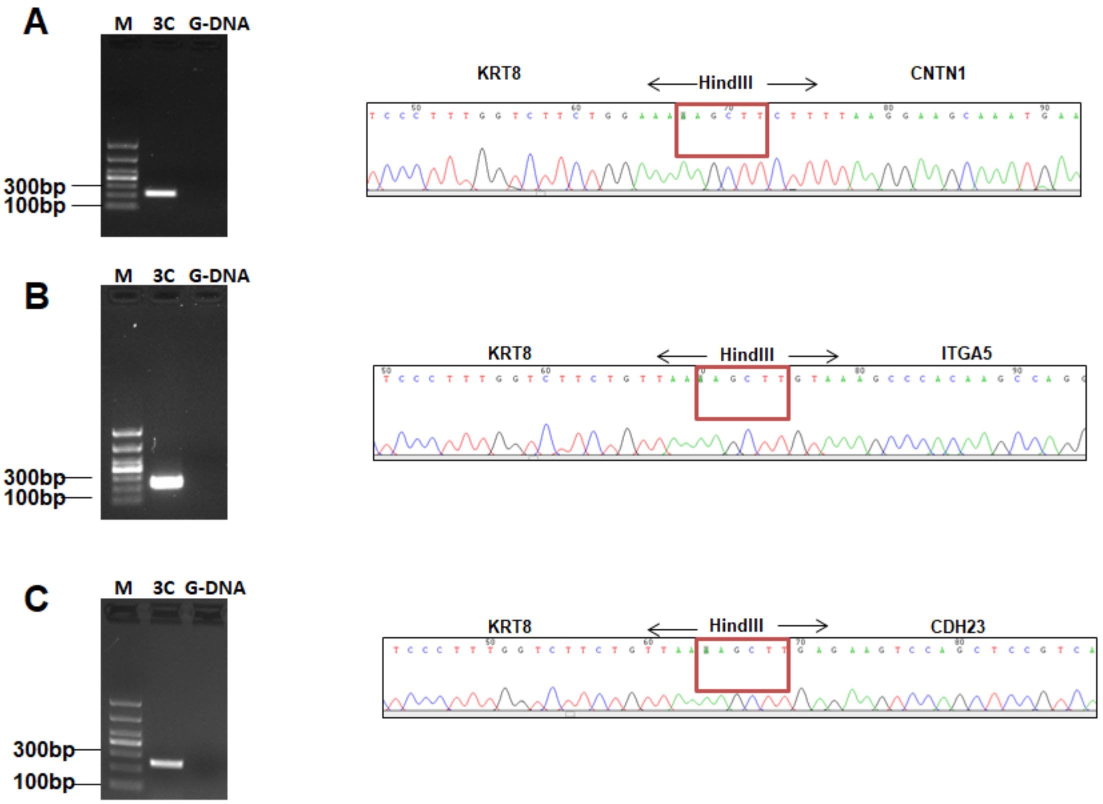

Fig. S7. 3C-PCR analysis confirms interaction of the rs55958994-associated enhancer and target gene loci. Interactions of the enhancer with the *CNTN1* (A), *ITGA5* (B) or *CDH23* (C) genes were verified by 3C-PCR and sequencing. M: marker; 3C: PCR products from the DNA fragments in the 3C library. Forward and reverse PCR primers were designed based on the rs55958994-associated enhancer region (KRT8) and target gene regions, respectively. G-DNA: Genomic DNA as the PCR template.

A

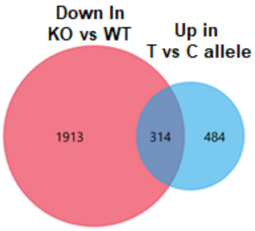

B

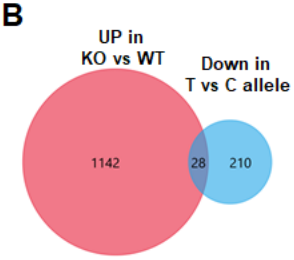

C

Gene expression changes

Symbol	fold change (WTvsKO)	Padj (KOvsWT)	fold change (TvsC)	Padj (TvsC)
CNTN1	59.18	9.71E-64	2.44	0.0001
KRT8	24.16	4.23E-37	0.58	0.0247
FAIM2	61.07	7.86E-49	1.68	0.1189
KRT7	15.64	6.89E-23	1.43	0.3344
ITGA5	2.78	1.75E-21	1.26	0.0591
KRT18	3.13	1.31E-15	0.8	0.1780
CDH23	4.85	4.72E-18	1.58	0.0594
SIPA1	2.74	1.45E-15	1.02	0.9063

Fig. S8. Comparison of transcriptomic changes following deletion of the rs55958994-associated enhancer and mutation of rs55958994. (A) Number of down-regulated genes in enhancer knock-out (KO) vs wild-type (WT) cell lines and up-regulated genes in risk allele T containing vs non risk C allele containing cell lines (fold-changes over1.5 and p-adjusted value <0.1). (B) Number of up-regulated genes in enhancer knock-out (KO) vs wild-type (WT) cell lines and down-regulated genes in risk allele T containing vs non risk C allele containing cell lines (fold-change over1.5 and p-adjusted value <0.1). (C) Comparison of 8 target genes' transcription changes following deletion of the rs55958994-associated enhancer and mutation of rs55958994.

Table S1. Guide RNAs for CRISPR-Cas9–mediated deletions.

gRNA	Sequence	Location in chromatin (hg19)
gRNA-1	TTCCACCTGCAATTACCTGATGG	chr12:53300416-53300438
gRNA-2	TTTCCCCTGACCCCTTATGCTGG	chr12:53303015-53302993

Table S2. Primers used in qRT-PCR experiment.

Gene name	Primer	Sequence
CNTN1	Forward	CAGCCCTTTCCCGGTTTACAA
	Reverse	TGCTTCTGACCATCCCGTAGT
KRT8	Forward	TCCTCAGGCAGCTATATGAAGAG
	Reverse	GGTTGGCAATATCCTCGTACTGT
FAIM2	Forward	AGTTCGTCGAGTCTTTGTCAGA
	Reverse	TGGGTCCAGAACAGCAAGC
KRT7	Forward	TGGGAGCCGTGAATATCTCTGT
	Reverse	GAGAAGCTCAGGGCATTGCT
ITGA5	Forward	GGCTTCAACTTAGACGCGGAG
	Reverse	TGGCTGGTATTAGCCTTGGGT
KRT18	Forward	GGCATCCAGAACGAGAAGGAG
	Reverse	ATTGTCCACAGTATTTGCGAAGA
CDH23	Forward	GCCTCTCGCTTCTTTGCAGT
	Reverse	AGGGCTGATTGTGAAATGTGG
SIPA1	Forward	GGAGCCACAGAACCGAACC
	Reverse	ACTCGTCCATCCCGAAGAAGT
HPRT1	Forward	CCTGGCGTCGTGATTAGTGAT
	Reverse	AGACGTTCAGTCCTGTCCATAA

Table S3. Primers for 3C-PCR assay.

primer	Sequence	Location in chromatin (hg19)
KRT8-3C-Forward	TTTCCAGGGCTGCCATACCCG	chr12:53301164-53301144
CNTN1-3C-Reverse	CCTGTCTAGTGTCTGCTTCTAAA	chr12: 41309232-41309210
ITGA5-3C-Reverse	CAGGAATAAGCCATGGGTAGTG	chr12:54807940-54807919
CDH23-3C-Reverse	CTTTCTGACCGGTCCTCTTTAC	chr10:73490704-73490683

Table S4. Guide RNAs for CRISPR-Cas9–mediated SNP editing.

gRNA	Sequence	Location in chromatin (hg19)
gRNA-1	AGTTAGCACCTATTTACACCAGG	chr12:53300927-53300949
gRNA-2	GCTCCCTCTAACAGCAGCGTGGG	chr12:53301197-53301219
SNP-F	CTGCCTCCTGCTATGCTGAA	Chr12:53300890-53300909
SNP-R	CCTCCCTCCTTCCGCCTTCC	Chr12:53301109-53301128
